# Pharmacological effects of berberine on models of ulcerative colitis: A meta-analysis and systematic review of animal studies

**DOI:** 10.3389/fphar.2022.937029

**Published:** 2022-09-06

**Authors:** Shuangyuan Hu, Pengfei Wei, Wei Li, Qingsong Liu, Shuanglan Chen, Caiyu Hu, Xiaochuan Guo, Xiao Ma, Jinhao Zeng, Yi Zhang

**Affiliations:** ^1^ Department of Gastroenterology, Hospital of Chengdu University of Traditional Chinese Medicine, Chengdu, China; ^2^ State Key Laboratory of Southwestern Chinese Medicine Resources, School of Pharmacy, Chengdu University of Traditional Chinese Medicine, Chengdu, China; ^3^ Department of Geriatrics, Hospital of Chengdu University of Traditional Chinese Medicine, Chengdu, China; ^4^ TCM Regulating Metabolic Diseases Key Laboratory of Sichuan Province, Hospital of Chengdu University of Traditional Chinese Medicine, Chengdu, China

**Keywords:** berberine, ulcerative colitis, animal model, meta-analysis, systematic review

## Abstract

Berberine (BBR) is the main active constituent of the *Rhizoma coptidis* (Huanglian) and has multiple biological activities. Although current evidence suggests that the BBR has a multi-target effect in ulcerative colitis (UC), its action and mechanism are unclear. The purpose of this meta-analysis was to assess the pharmacological effects and potential mechanisms of BBR in UC models. Studies were searched from four databases (PubMed, Embase, Web of Science, and Cochrane Library) until March 2022. Standardized mean difference (SMD) and 95% confidence intervals (CI) were used for the adjudication of outcomes. Stata 15.0 software was used for statistical analysis. Twenty-eight publications and 29 studies involving 508 animals were included in the meta-analysis. The results showed that BBR reduced disease activity index (DAI) scores, alleviated UC-induced colon length (CL) loss, prevented weight loss, and reduced histological colitis score (HCS). Mechanistically, BBR was found to reduce myeloperoxidase (MPO) activity and malondialdehyde (MDA) levels, reduce levels of pro-inflammatory factors interleukin-1β (IL-1β), interleukin 6 (IL-6), tumor necrosis factor α (TNF-α), interferon-γ (IFN-γ) and mRNA expression of interleukin 17, increase levels of anti-inflammatory factor interleukin 10 (IL-10), and to increase levels of tight junction protein zonula occludens-1 (ZO-1) and occludin, which may involve antioxidant, anti-apoptotic, neuromodulation, anti-fibrotic, anti-inflammatory, barrier protection, and flora regulation aspects. However, additional attention should be paid to these outcomes due to the heterogeneity and methodological quality of the studies.

## 1 Introduction

Ulcerative colitis (UC) is a form of inflammatory bowel disease (IBD) (Ford et al., 2013). IBD, including UC and Crohn’s disease, has a clear association with the development of colorectal cancer (Macarthur et al., 2004). UC has been recognized as a global disease because the incidence is steadily increasing worldwide (Pravda, 2019). UC is characterized by spontaneous chronic, relapsing-remitting inflammation of the colon, typically presenting as bloody diarrhoea and chronic pain (Abraham and Cho, 2009). The exact cause of UC remains unknown. Current studies have shown that abnormal activation of the immune system, hereditary susceptibility, and alteration of intestinal flora caused by mucosal barrier defects may play a role in the pathophysiology of UC (Khor et al., 2011; Steel et al., 2011; Kostic et al., 2014). Traditional IBD medication aims to control the immune response with corticosteroids, aminosalicylates, and immunosuppressants (Podolsky, 2002). As these drugs show limited efficacy, variable responses, and strong side effects, efforts have been made in recent years to develop novel therapy options (Rutgeerts et al., 2009). The discovery of therapeutic drugs with clear efficacy and low side effects is the difficult part of UC treatment. Natural products and their corresponding derivatives are of great interest to the pharmaceutical industry and have given rise to many scientific studies (Ekiert and Szopa, 2020).

**FIGURE 1:**
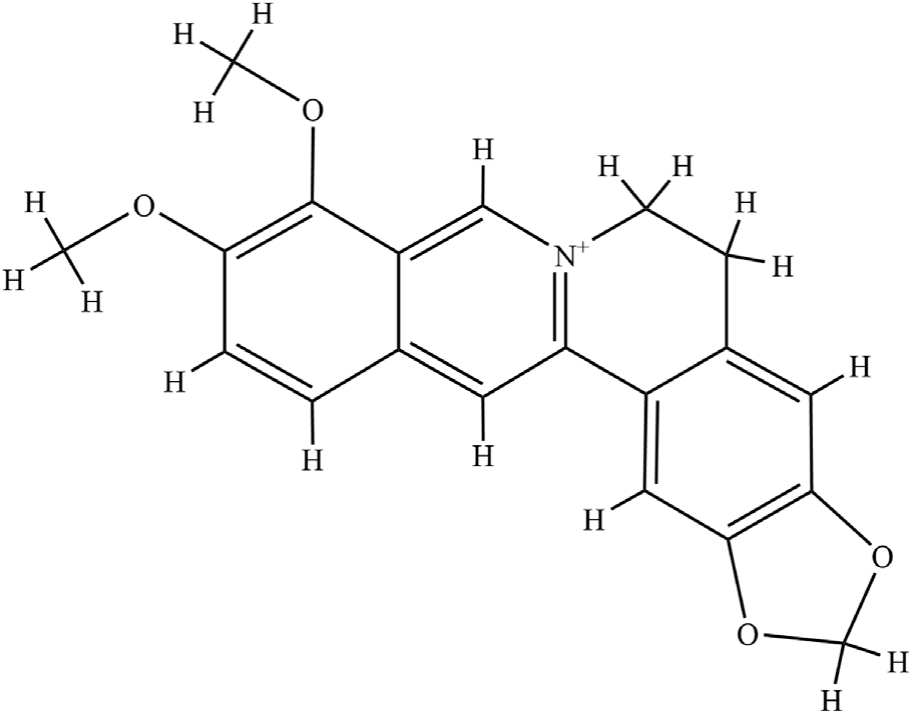
The chemical structure of berberine.

Berberine (BBR, C_20_H_18_NO_4_^+^, Figure 1) is a yellow crystalline isoquinoline alkaloid derived from a variety of plants, such as *Hydrastus canadensis*, *Pellodendron chenins*, and *Rhizoma coptidis* (Huanglian) (Meng et al., 2018; Wang et al., 2019). BBR has been used for centuries in Chinese traditional medicine and Indian medicine (Cordell et al., 2001; Imanshahidi and Hosseinzadeh, 2008). Studies have shown that BBR has several pharmacological activities such as antidepressant, antitypical diabetes, and antitumor effects (Sun et al., 2014; Zhou et al., 2019; Rauf et al., 2021). However, due to the limited clinical studies of BBR, especially for UC treatment, there is no clear clinical evidence. Many experimental studies showed the beneficial role of BBR in UC treatment. Therefore, understanding the role and mechanism of BBR in the treatment of UC in animal models is of great significance to the potential clinical application of BBR.

Researchers using animals for experiments are being tested ethically and morally (Meier and Stocker, 1989). Integrating the results of existing animal experiments could enhance the reliability of conclusions while reducing unnecessary animal sacrifice (Locker, 2004; Levy, 2012; Ioannidis et al., 2014). Constructing evidence-based evidence at the animal level can help translate preclinical findings into the clinic, such as meta-analyses of cannabinoids, and endogenous cannabinoids on pain in animal models (Finn et al., 2021). Although the available *in vivo* and *in vitro* studies suggest that BBR has great potential in the treatment of UC, its pharmacological effects and safety remain controversial. In particular, to date, no meta-analysis based on preclinical studies has been performed to synthesize the role of BBR in UC. To investigate the potential effects of BBR on UC and expand the understanding of the synergistic effects of BBR for UC, we used animal meta-analysis to construct preclinical evidence and provide systematic scientific support for further clinical studies of BBR.

## 2 Methods

The Preferred Reporting Items for Systematic Reviews and Meta-Analysis (PRISMA) were followed to design and conduct the current systematic review and meta-analysis (Page et al., 2021).

### 2.1 Literature search

To obtain comprehensive information on preclinical studies of BBR in the treatment of UC, studies were searched from four databases (PubMed, Embase, Web of Science, and Cochrane Library). The search was conducted until 5 March 2022. The participating authors discussed search methods to minimize the loss of research literature. Finally, an approach combined with disease and treatment was used. In PubMed, searches were performed using Mesh word search with search terms such as “ulcerative colitis” “Colitis Gravis” and “Berberine” “Dioxolanes” (Supplemental Table S1).

### 2.2 Inclusion criteria

1) Results of published studies. 2) Studies with separate treatment and control groups were available. 3) There are no restrictions on animal modeling methods, animal species, animal sex, size, or sample size. 4) The experimental group was only treated with BBR, while the model group was treated with vector or as model control. 5) The experimental data can be obtained.

### 2.3 Exclusion criteria

1) Reviews, case reports, clinical studies, and *in vitro* experiments. 2) Preclinical studies that were unrelated to UC. 3) Repeatedly published literature. 4) Experimental data in the literature were not available. 5) Experimental data with significant bias.

### 2.4 Data extraction

The following information was extracted independently by two authors: 1) The authors of the literature and the year of publication. 2) The species, sex, weight range, and sample size of the animals. 3) The modeling method of the animal model of UC. 4) The type of anesthetic drugs used. 5) Interventions in the model and treatment groups, intervention period, and intervention dose. 6) Outcome indicators: histological colitis score (HCS), body weight change (BWC), colon length (CL), disease activity index (DAI), mRNA expression of interleukin 17, interleukin 6 (IL-6), interleukin-1β (IL-1β), interferon-γ (IFN-γ), interleukin 10 (IL-10), tumor necrosis factor α (TNF-α), zonula occludens-1 (ZO-1), occludin, myeloperoxidase (MPO), malondialdehyde (MDA), phospholipase A2-IVA (PLA2G4A). When there were multiple groups in the same experiment, the group with the highest dose was selected. For data in graphical form, raw data were first sought from the authors, otherwise, data were obtained by electronic data calipers. If the data in the text are presented as standard error (SEM), it will be converted into standard deviation (SD), and the formula is SD = SEM * n1/2 (Lee et al., 2015).

### 2.5 Quality evaluation

Two authors independently evaluated the risk of bias using the 10-item scale of the Center for the Evaluation of Laboratory Animal Experiments (SYRCLE) risk (Hooijmans et al., 2014) bias tool to evaluate the quality of the literature on BBR for UC. The entries for quality evaluation are as follows: selectivity bias, implementation bias, measurement bias, missed visit bias, reporting bias, and other biases. Any divergences in the process of quality assessment were finally resolved by consultation with the correspondence author.

### 2.6 Data analysis

The statistical analysis was performed using STATA software version 15.0. Since the outcome indicators in this paper are continuous variable type data, the outcomes were judged using standardized mean difference (SMD) and 95% confidence intervals (CI). *p* < 0.05 for the outcome indicators indicated a significant difference between the experimental group and the model group. Heterogeneity was evaluated using I-square (I^2^) as an index. I^2^ ≤ 50% indicated that the heterogeneity of the included studies was small and the effect sizes of the outcomes were combined using a fixed-effects model. I^2^ > 50% indicated that the heterogeneity of the included studies was large, and a random-effects model was used to combine the effect sizes of the results. Subgroup analysis was performed on data included in studies >10 and with large heterogeneity in outcomes to explore possible sources of heterogeneity, and we pre-established subgroups for species, sex, treatment cycles (≤10 days and >10 days), and treatment dose (≤50 mg/kg and >50 mg/kg). Potential publication bias was assessed using Egger’s test if the number of included datasets was 10 or higher. And the trim and fill method was performed in the presence of publication bias. To better demonstrate the effect of dose and time of administration on the results, time-dose effect relationship plots were created. When multiple groups were involved in the same study, groups that achieved a significant difference were included in the time-dose analysis.

## 3 Results

### 3.1 Study inclusion

**FIGURE 2:**
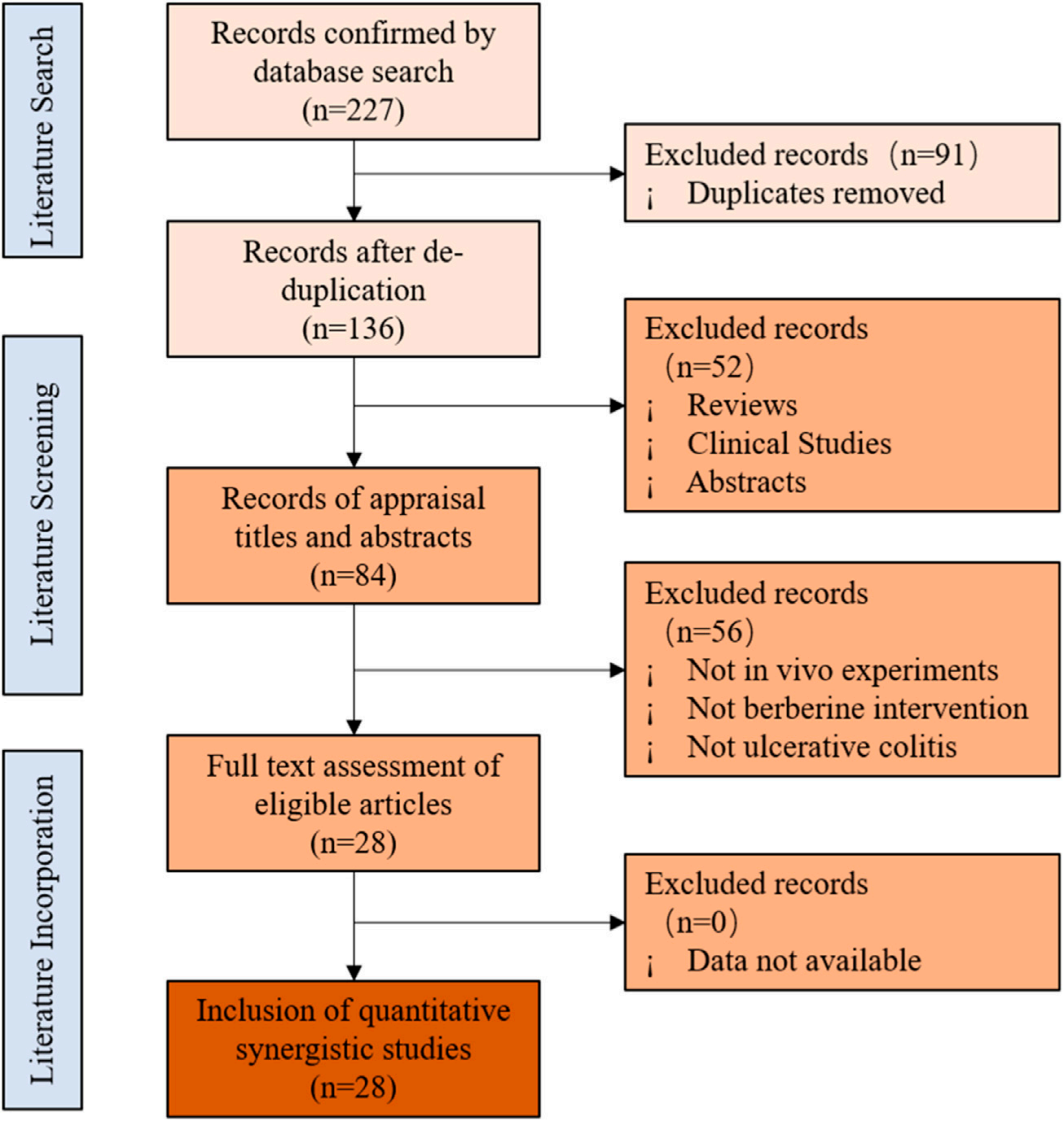
Flowchart of selection for studies inclusion.

A database search identified 227 potentially relevant articles, including 62 from PubMed, 66 from Web Science, 94 from Embase, and 5 from the Cochrane library. After incorporating all searches and getting rid of duplicates, 136 records were retained. Of the remaining articles, 52 records were removed by reading the titles and abstracts. Finally, 28 articles were finally included after full text assessment (Hong et al., 2012; Li et al., 2016a; Li et al., 2016b; Zhang et al., 2017; Cui et al., 2018; Jing et al., 2018; Li and Shen, 2018; Yu et al., 2018; Li et al., 2019a; Li et al., 2019b; Hui et al., 2019; Liao et al., 2019; Zhu et al., 2019; Jia et al., 2020a; Li et al., 2020a; Jia et al., 2020b; Li et al., 2020b; Deng et al., 2020; Ding et al., 2020; Han et al., 2020; Liao et al., 2020; Yan et al., 2020; Zhai et al., 2020; Li et al., 2021a; Li et al., 2021b; Jiang et al., 2021; Zheng et al., 2021; Yang et al., 2022). The document screening flow chart is shown in Figure 2. All included studies were published in the last decade (2012–2021), indicating that the protective effect of BBR on UC has attracted strong interest in recent years.

### 3.2 Characteristics of the included studies

Twenty-eight articles including 29 studies published in English were included. A total of 508 animals were included, including 254 in the experimental group and 254 in the model group. Animal species included BALB/c mice (Hong et al., 2012; Cui et al., 2018; Li and Shen, 2018; Yu et al., 2018; Li et al., 2019a; Jia et al., 2020a; Deng et al., 2020; Yan et al., 2020; Li et al., 2021a; Li et al., 2021b), C57BL/6 mice (Li et al., 2016a; Li et al., 2016b; Zhang et al., 2017; Li et al., 2019b; Li et al., 2020a; Li et al., 2020b; Ding et al., 2020; Han et al., 2020; Zhai et al., 2020; Yang et al., 2022), kunming mice (Hui et al., 2019), Wistar rats (Zhu et al., 2019; Jia et al., 2020b), and Sprague-Dawley rats (Jing et al., 2018; Liao et al., 2019; Liao et al., 2020; Jiang et al., 2021). One study (Zheng et al., 2021) indicated the animals were mice, instead of mentioning their specific strain. Twenty papers used only male animals to conduct experiments (Li et al., 2016a; Li et al., 2016b; Zhang et al., 2017; Jing et al., 2018; Li and Shen, 2018; Yu et al., 2018; Li et al., 2019b; Hui et al., 2019; Liao et al., 2019; Zhu et al., 2019; Jia et al., 2020a; Li et al., 2020a; Jia et al., 2020b; Li et al., 2020b; Han et al., 2020; Liao et al., 2020; Yan et al., 2020; Zhai et al., 2020; Li et al., 2021a; Li et al., 2021b). Five papers used only females for the experiments (Hong et al., 2012; Cui et al., 2018; Li et al., 2019a; Ding et al., 2020; Yang et al., 2022). Three papers did not mention animal sex (Deng et al., 2020; Jiang et al., 2021; Zheng et al., 2021). Regarding the use of anesthetics in animals, three papers used chloral hydrate as an anesthetic (Li et al., 2016b; Zhang et al., 2017; Zhai et al., 2020), and one paper used sodium pentobarbital as an anesthetic (Jiang et al., 2021), two studies used ether as an anesthetic (Yu et al., 2018; Jia et al., 2020b), and one paper used anesthesia but did not mention the specific anesthetic (Jing et al., 2018). The rest of the literature is silent on the use of anesthetics in experiments. In terms of animal modeling, one paper used an intra-rectal injection of acetic acid for UC modeling (Jia et al., 2020b), and the rest used sodium dextran sulfate added to drinking water. The animal models were screened for the pathological stage of UC. The experimental group was treated with BBR and the model group was given a vector or blank control. To evaluate the effect of BBR in the treatment of UC, the included literature was compiled. Eight studies reported the rate of weight change. Eighteen studies reported DAI. Nineteen studies reported CL. Twenty-four studies reported histopathological analysis. Four studies reported IL-10 levels. Eight studies reported IL-1β levels. Nine studies reported IL-6 levels. Five studies reported IFN-γ levels. Three studies reported IL-17 mRNA expression levels. Thirteen studies reported MPO activity. Five studies reported ZO-1 levels. Five studies reported occludin levels. Two studies reported the MDA levels. One study reported the level of PLA2G4A. Detailed information on BBR in each study is displayed in Table 1. The details of the included studies are presented in Table 2.

**TABLE 1:** Information of berberine of each study.

Study (years)	Source	Purity (%)	Quality control reported
Hong et al. (2012)	Unknown	Unknown	Unknown
Li et al. (2016a)	Zelang Group (Nan- jing, China)	Unknown	Unknown
Li et al. (2016b)	Shenzhen ChemStrong Scientific Co., Ltd (Shenzhen, China)	>95%	HPLC
Zhang et al. (2017)	Sigma-Aldrich (Merck KGaA, Darmstadt, Germany)	Unknown	Unknown
Cui et al. (2018)	Solarbio Biotechnology Co., Ltd. (Beijing, China)	>98%	HPLC
Jing et al. (2018)	Shanghai Boyun Biotech Co., Ltd. (Shanghai, China)	>95%	Unknown
Li and Shen, (2018)	Sigma-Aldrich (St. Louis, MO, United States)	>95%	HPLC
Yu et al. (2018)	Xi’an Realin Biotechnol-ogy Co. Ltd. (Xi’an, China)	>98%	HPLC
Hui et al. (2019)	Chenguang biotech Group Co., Ltd (Shanxi, China)	>98%	HPLC
Li et al. (2019a)	National Institutes for Food and Drug Control (Beijing, China)	>95%	Unknown
Li et al. (2019b)	Shenzhen ChemStrong Scientific Co., Ltd (Shenzhen, China)	>95%	Unknown
Liao et al. (2019)	Dalian Meilun Biotechnology Co., Ltd (Dalian, China)	>98%	HPLC
Zhu et al. (2019)	Sigma-Aldrich (Merck Millipore, Darmstadt, Germany)	Unknown	Unknown
Deng et al. (2020)	Yuanye Bio-Technology (Shanghai, China)	>95%	HPLC
Ding et al. (2020)	Sigma-Aldrich (St. Louis, MO, United States)	>95%	HPLC
Han et al. (2020)	Shenzhen ChemStrong Scientific Co., Ltd (Shenzhen, China)	>95%	HPLC
Jia et al. (2020a)	Sigma - Aldrich (Shanghai; China)	>98%	Unknown
Jia et al. (2020b)	Sigma-Aldrich (St. Louis, MO, United States)	>95%	HPLC
Li et al. (2020a)	Sigma-Aldrich (St. Louis, MO, United States)	>95%	HPLC
Li et al. (2020b)	Sigma-Aldrich (St. Louis, MO, United States)	>95%	HPLC
Yan et al. (2020)	Yuanye Bio-Technology (Shanghai, China)	>95%	HPLC
Liao et al. (2020)	Unknown	Unknown	Unknown
Zhai et al. (2020)	Shenzhen ChemStrong Scientific Co., Ltd (Shenzhen, China)	>95%	HPLC
Jiang et al. (2021)	Shanghai Xinyi Tianping Pharmaceutical Co., Ltd	Unknown	Unknown
Li et al. (2021a)	Chengdu Herb-purify Co., Ltd (Chengdu, China)	>98%	HPLC
Li et al. (2021b)	Unknown	Unknown	Unknown
Zheng et al. (2021)	Unknown	Unknown	Unknown
Yang et al. (2022)	Unknown	Unknown	Unknown

**TABLE 2:** Basic characteristics of the included studies.

Study (year)	Species (sex, *n* = BBR/model group, weight)	Model method	BBR group (administration, drug dose, duration)	Model group (administration, drug dose, duration)	Outcome index
Hong et al. (2012)	BALB/c mice (female, 8/8)	5% DSS in the drinking water (10 days)	By gavage, 40 mg/kg/d, 10 days	By gavage, distilled water, 10 days	③⑪⑭
Li et al. (2016a)	C57BL/6 mice (male, 10/10, 18–22 g)	5% DSS in the drinking water (7 days)	By gavage, 100 mg/kg/d, 7 days	By gavage, 0.5%CMC-Na, 7 days	②③④
Li et al. (2016b)	C57BL/6 mice (male, 8/8)	2% DSS in the drinking water (15 days)	By gavage, 20 mg/kg/d, 30 days	By gavage, drinking water, 30 days	②③④⑩⑫⑬
Zhang et al. (2017)	C57BL/6 mice (male, 10/10, 18–22 g)	5% DSS in the drinking water (6 days)	By gavage, 100 mg/kg/d, 5 days	No mention	③④⑪⑫⑬
Cui et al. (2018)	BALB/c mice (female, 10/10, 18–22 g)	4%DSS in the drinking water (7 days)	By gavage, 40 mg/kg/d, 10 days	By gavage, normal saline, 10 days	①②③④⑤⑩
Jing et al. (2018)	Sprague-Dawley rats (male, 8/8, about 250 g)	5% DSS in the drinking water (7 days)	By gavage, 40 mg/kg/d, 7 days	By gavage, drinking water, 7 days	②⑥⑦⑧⑪
Li and Shen, (2018)	BALB/c mice (male, 12/12, 18–22 g)	5% DSS in the drinking water (14 days)	By gavage, 100 mg/kg/d, 7 days	By gavage, distilled water, 7 days	②④⑫⑬
Yu et al. (2018)	BALB/c mice (male, 10/10)	3% DSS in the drinking water (7 days)	By gavage, 50 mg/kg/d, 7 days	By gavage, 0.5%CMC-Na, 7 days	②③④⑥⑦⑧⑨⑪⑫⑬
Hui et al. (2019)	KM mice (male, 10/10, 15–25 g)	2% DSS in the drinking water (15 days)	By gavage, 100 mg/kg/d, 20 days	By gavage, normal saline, 20 days	④
Li et al. (2019a)	BALB/c mice (female, 7/7)	2% DSS in the drinking water (7 days)	By gavage, 50 mg/kg/d, 7 days	By gavage, distilled water, 7 days	②④
Li et al. (2019b)	C57BL/6 mice (male, 5/5)	2% DSS in the drinking water (12 days)	40 mg/kg/d, 14 days	drinking water, 14 days	③④
Liao et al. (2019)	Sprague-Dawley rats (male, 8/8, 160–180 g)	4% DSS in the drinking water (7 days)	By gavage, 100 mg/kg/d, 6 days	By gavage, 0.5%CMC-Na, 6 days	②③④⑤⑦⑧
Zhu et al. (2019)	Wistar rats (male, 10/10, 200–230 g)	5% DSS in the drinking water (7 days)	By gavage, 50 mg/kg/d, 49 days	No mention	②③④⑪
Deng et al. (2020)	BALB/c mice (6/6, 20–24 g)	2.5% DSS in the drinking water (7 days)	By gavage, 20 mg/kg/d, 4 days	By gavage, Drinking water, 4 days	②③④⑪
Ding et al. (2020)	C57BL/6 mice (female, 6/6)	3% DSS in the drinking water (7 days)	By gavage, 50 mg/kg/d, 10 days	By gavage, normal saline, 10 days	①②③⑥⑦⑧⑪
Han et al. (2020)	C57BL/6 mice (male, 4/4, 23–25 g)	2% DSS in the drinking water (12 days)	By gavage, 40 mg/kg/d, 14 days	By gavage, drinking water, 14 days	①②③⑩
Han et al. (2020)	C57BL/6 mice (male, 6/6, 23–25 g)	2% DSS in the drinking water (15 days)	By gavage, 40 mg/kg/d, 30 days	By gavage, drinking water, 30 days	①②③④
Jia et al. (2020a)	BALB/c mice (male, 8/8, 18–22 g)	5% DSS in the drinking water (7 days)	By gavage, 50 mg/kg/d, 14 days	By gavage, pure water, 14 days	②③④⑪
Jia et al. (2020b)	Wistar rats (male, 7/7, 200–225 g)	intrarectal injection of 2 ml acetic acid (AcOH, 4%)	By gavage, 50 mg/kg/d, 7 days	By gavage, normal saline, 7 days	④⑥⑦⑧⑪
Li et al. (2020a)	C57BL/6 mice (male, 8/8, 23–25 g)	3% DSS in the drinking water (7 days)	By gavage, 100 mg/kg/d, 10 days	By gavage, sterile water, 10 days	①②③④⑤⑥⑦⑧⑨⑪⑫⑬⑭
Li et al. (2020b)	C57BL/6 mice (male, 15/15, 22–24 g)	2% DSS in the drinking water (21 days)	By gavage, 50 mg/kg/d, 35 days	By gavage, sterile water, 35 days	①②③④⑥⑦⑧⑨
Yan et al. (2020)	BALB/c mice (male, 12/12, 18–22 g)	5% DSS in the drinking water (7 days)	By gavage, 200 mg/kg/d, 7 days	By gavage, distilled water, 7 days	④
Liao et al. (2020)	Sprague-Dawley rats (male, 8/8, 160–180 g)	4% DSS in the drinking water (7 days)	By gavage, 100 mg/kg/d, 6 days	By gavage,0.5%CMC-Na, 6 days	④
Zhai et al. (2020)	C57BL/6 mice (male, 10/10)	2% DSS in the drinking water (3 days)	By gavage, 20 mg/kg/d, 4 days	By gavage, distilled water, 4 days	⑮
Jiang et al. (2021)	Sprague-Dawley rats (20/20, 210–230 g)	1.5%–2% DSS in the drinking water (17days)	By gavage, 30 mg/kg/d, 17 days	By gavage, normal saline, 17 days	④⑤⑥⑦⑧
Li et al. (2021a)	BALB/c mice (male, 10/10, 24–26 g)	3% DSS in the drinking water (8 days)	By gavage, 50 mg/kg/d, 9 days	By gavage, drinking water, 9 days	②③④⑥⑦⑧⑨⑪
Li et al. (2021b)	BALB/c mice (male, 6/6, 15–20 g)	4% DSS in the drinking water (7 days)	By gavage, 150 mg/kg/d, 14 days	By gavage, distilled water, 14 days	②④⑧⑨⑪
Zheng et al. (2021)	Mice (6/6)	3% DSS in the drinking water (5 days)	By gavage, 100 mg/kg/d, 8 days	By gavage, Phosphate-buffered saline, 8 days	①③④
Yang et al. (2022)	C57BL/6 mice (female, 6/6)	2.5% DSS in the drinking water (7 days)	By gavage, 40 mg/kg/d, 7 days	No mention	①③④⑪

Note: ①BWC ②DAI ③CL ④HA ⑤IL-10 ⑥IL-1β ⑦IL-6 ⑧TNF-α ⑨IFN-γ ⑩mRNA expression levels of IL-17 ⑪MPO ⑫ZO-1 ⑬occludin ⑭MDA ⑮PLA2G4A.

### 3.3 Study quality

An evaluation of the quality of the literature included in this paper showed that twenty-four of the twenty-nine studies reported using a randomized approach to grouping animals but did not mention the specific randomization method, and five studies did not mention randomized grouping. All studies reported baseline characteristics between the groups. No studies have described whether the allocation of different groups is sufficiently concealed. The experimental setting was identical in each study, and therefore, we consider the placement of animals to be consistent with the principle of randomization. No studies mentioned the Blinding of experimentalists. Seven studies used randomized outcome assessments. Two studies described the use of blinding for outcome assessment. No studies described outcome data completeness, and none found selective reporting or other source bias. The methodological quality of included studies is displayed in Table 3.

**TABLE 3:** The methodological quality of included studies.

Study year	A	B	C	D	E	F	G	H	I	J	Total
Hong et al. (2012)	−	+	−	+	−	−	+	+	+	+	6
Li et al. (2016a)	−	+	−	+	−	+	−	+	+	+	6
Li et al. (2016b)	?	+	−	+	−	−	−	+	+	+	5
Zhang et al. (2017)	?	+	−	+	−	−	−	+	+	+	5
Li and Shen, (2018)	−	+	−	+	−	−	−	+	+	+	5
Yu et al. (2018)	−	+	−	+	−	+	−	+	+	+	6
Jing et al. (2018)	−	+	−	+	−	−	−	+	+	+	5
Cui et al. (2018)	?	+	−	+	−	+	−	+	+	+	6
Zhu et al. (2019)	?	+	−	+	−	−	−	+	+	+	5
Liao et al. (2019)	?	+	−	+	−	−	−	+	+	+	5
Li et al. (2019a)	?	+	−	+	−	−	−	+	+	+	5
Li et al. (2019b)	?	+	−	+	−	−	−	+	+	+	5
Hui et al. (2019)	?	+	−	+	−	−	−	+	+	+	5
Li et al. (2020a)	?	+	−	+	−	−	−	+	+	+	5
Li et al. (2020b)	?	+	−	+	−	−	+	+	+	+	6
Ding et al. (2020)	?	+	−	+	−	−	−	+	+	+	5
Yan et al. (2020)	?	+	−	+	−	−	−	+	+	+	5
Jia et al. (2020a)	?	+	−	+	−	−	−	+	+	+	5
Jia et al. (2020b)	?	+	−	+	−	−	−	+	+	+	5
Deng et al. (2020)	?	+	−	+	−	−	−	+	+	+	5
Zhai et al. (2020)	?	+	−	+	−	+	−	+	+	+	6
Liao et al. (2020)	?	+	−	+	−	−	−	+	+	+	5
Han et al. (2020)	?	+	−	+	−	−	−	+	+	+	5
Han et al. (2020)	?	+	−	+	−	−	−	+	+	+	5
Jiang et al. (2021)	?	+	−	+	−	−	−	+	+	+	5
Li et al. (2021a)	?	+	−	+	−	+	−	+	+	+	6
Li et al. (2021b)	?	+	−	+	−	+	−	+	+	+	6
Zheng et al. (2021)	?	+	−	+	−	−	−	+	+	+	5
Yang et al. (2022)	?	+	−	+	−	+	−	+	+	+	6

(A) Sequence generation. (B) Baseline characteristics. (C) Allocation concealment. (D) Random housing. (E) Blinding of experimentalists. (F) Random outcome assessment. (G) Blinding of outcome assessors. (H) Incomplete outcome data. (I) Selective outcome reporting. (J) Other sources of bias. +: indicates low risk; − indicates high risk; ? indicates unclear risk.

### 3.4 Effectiveness

#### 3.4.1 Histological colitis score

**FIGURE 3:**
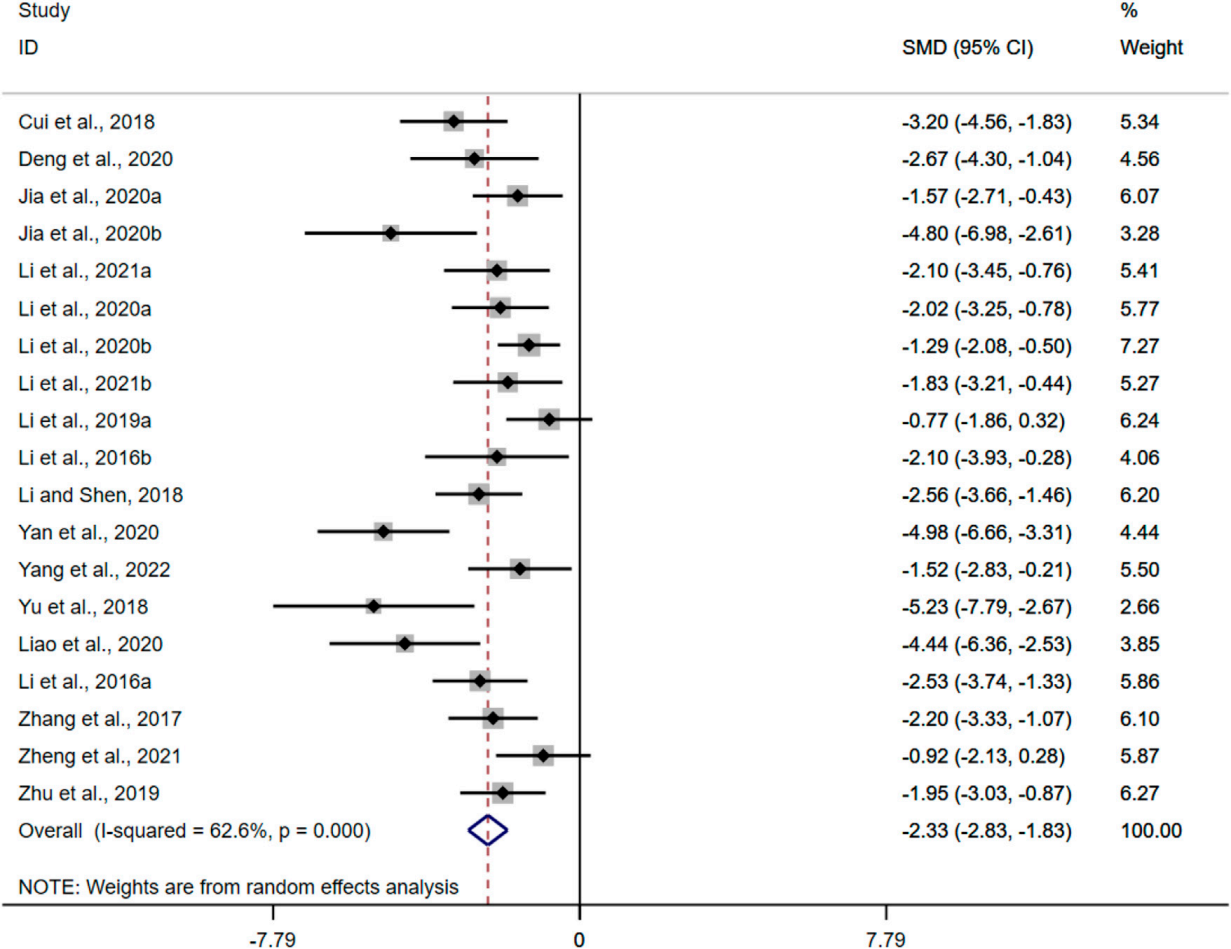
Forest plot: effect of berberine on HCS level.

Histopathological analysis was performed in twenty-four of the twenty-nine included studies, five of which used only H and E (hematoxylin and eosin) staining. Nineteen studies performed quantitative HCS based on H and E. The results of the overall analysis showed that BBR could reduce HCS levels in UC models [*n* = 316, SMD = −2.33, 95%CI (−2.83, −1.83), *p* < 0.05; Figure 3]. The heterogeneity test indicated the presence of heterogeneity (I^2^ = 62.6%, *p* < 0.01).

#### 3.4.2 Body weight change

**FIGURE 4:**
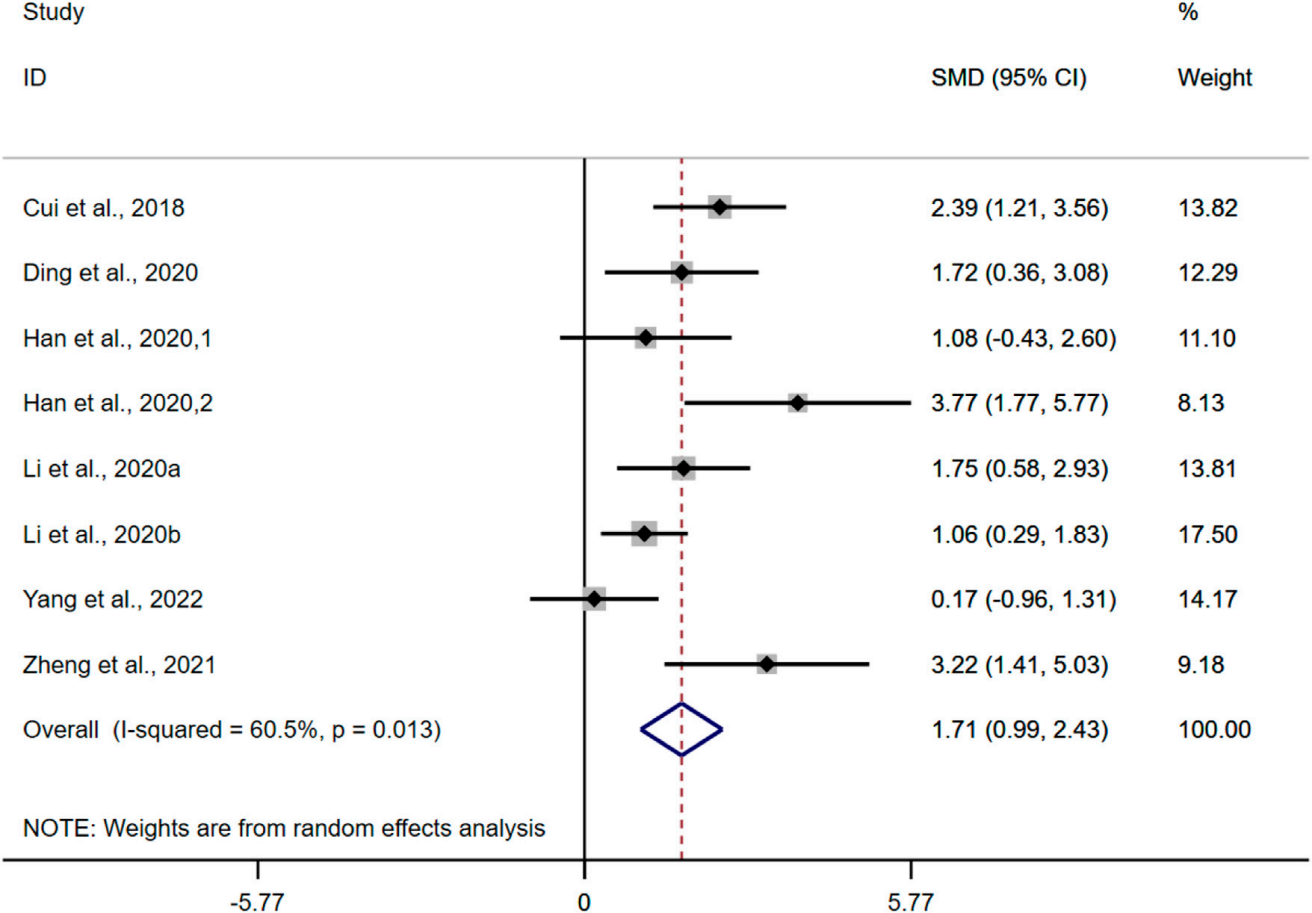
Forest plot: effect of berberine on BWC level.

According to the data from the eight studies, the results of the overall analysis showed that BBR could increase BWC levels in UC models [*n* = 122, SMD = 1.71, 95%CI (0.99, 2.43), *p* < 0.05; Figure 4]. The heterogeneity test indicated the presence of heterogeneity (I^2^ = 60.5%, *p* = 0.013).

#### 3.4.3 Disease activity index

**FIGURE 5:**
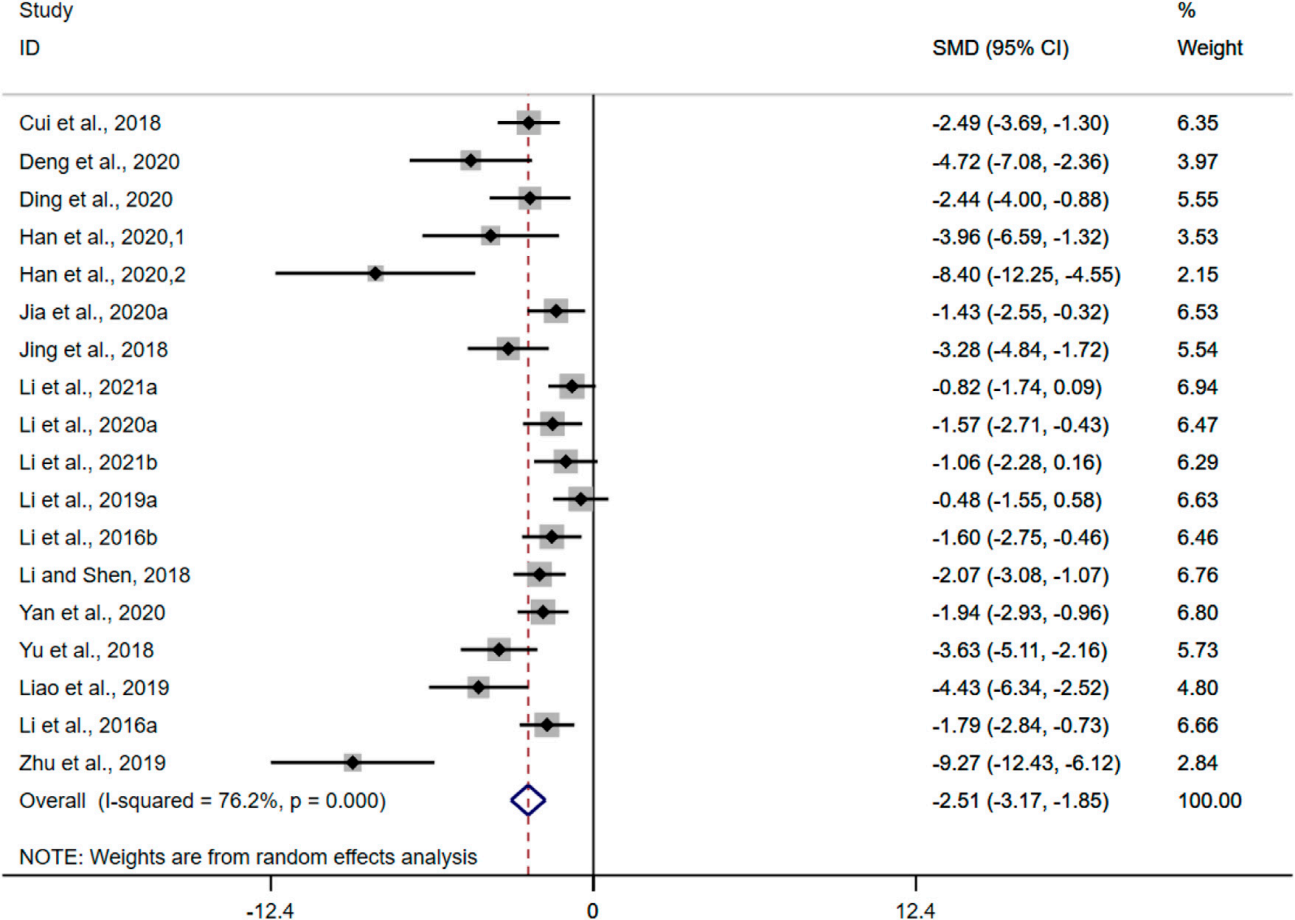
Forest plot: effect of berberine on DAI scores level.

DAI was adopted as the outcome measure in eighteen studies. The results of the overall analysis showed that BBR could reduce DAI levels in UC models [*n* = 298, SMD = −1.98, 95%CI (−2.28, −1.67), *p* < 0.05; Figure 5]. The heterogeneity test indicated the presence of heterogeneity (I^2^ = 76.2%, *p* < 0.01).

#### 3.4.4 Colon length

**FIGURE 6:**
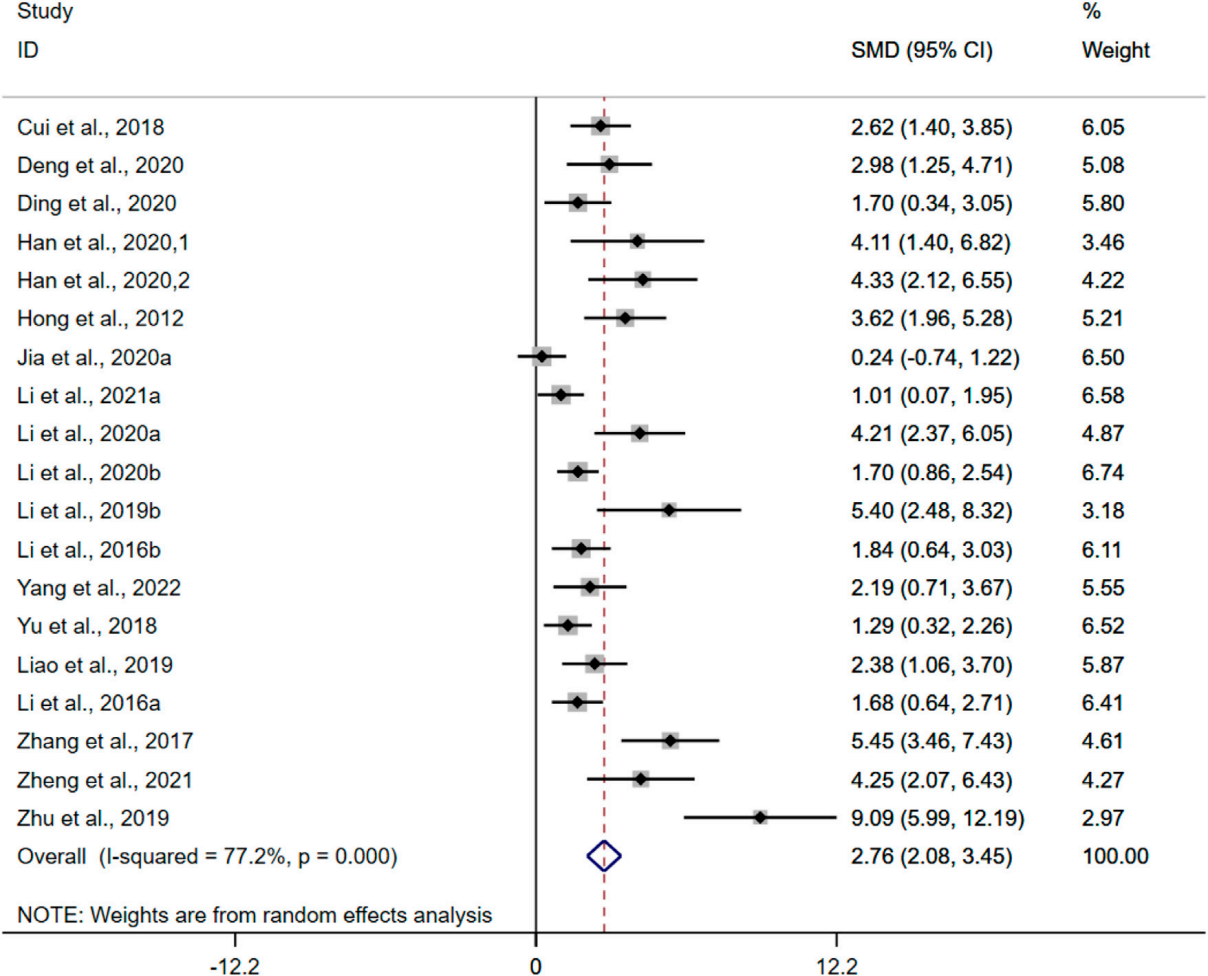
Forest plot: effect of berberine on CL level.

CL was adopted as the outcome measure in nineteen studies. The results of the overall analysis showed that BBR could increase CL levels in UC models [*n* = 308, SMD = 2.76, 95%CI (2.08, 3.45), *p* < 0.05; Figure 6]. The heterogeneity test indicated the presence of heterogeneity (I^2^ = 77.2%, *p* < 0.01).

#### 3.4.5 Inflammation-related indicators

##### 3.4.5.1 Interleukin 10

**FIGURE 7:**
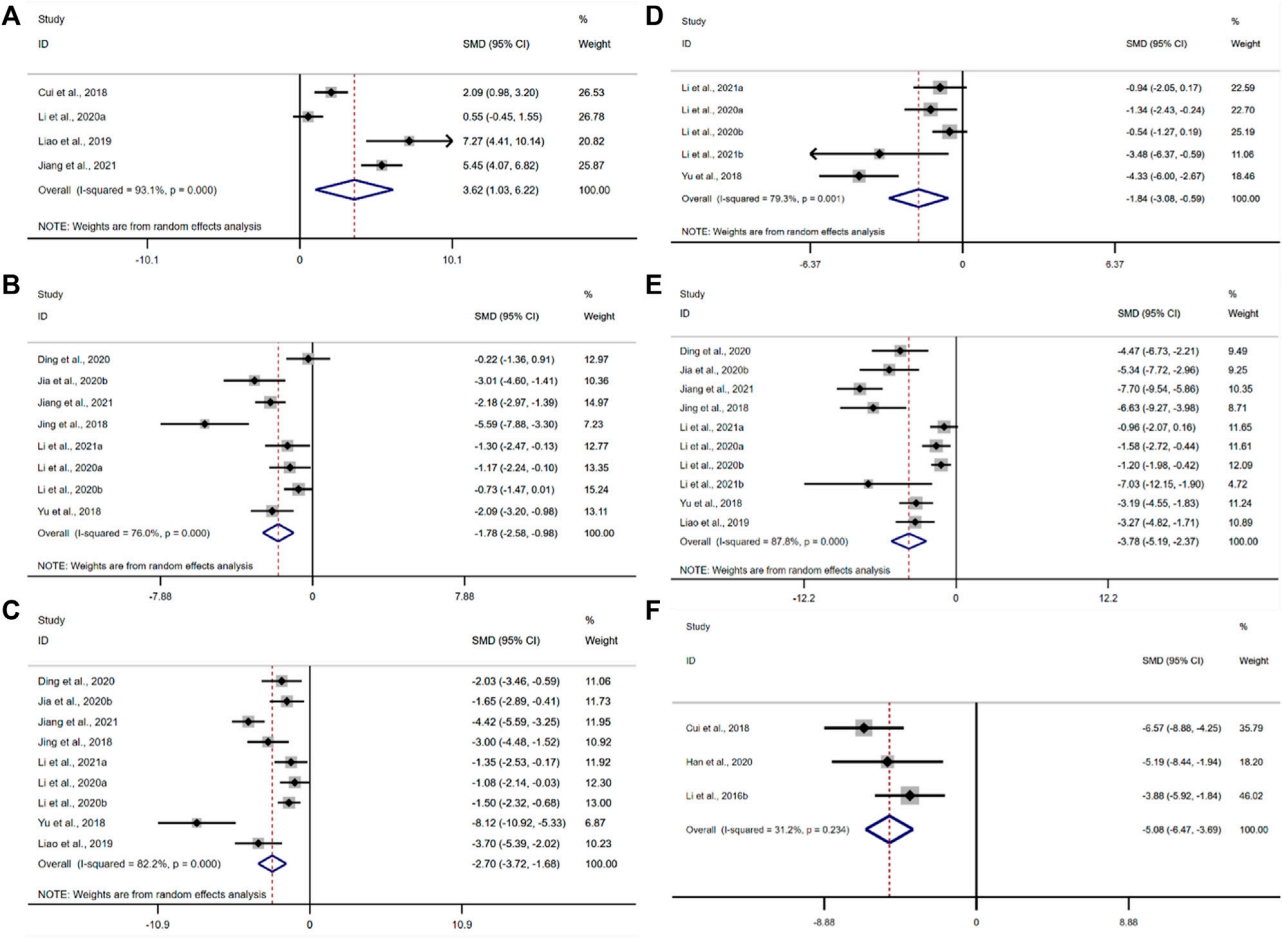
Forest plot: effect of berberine on **(A)** IL-10, **(B)** IL-1β, **(C)** IL-6, **(D)** IFN-γ, **(E)** TNF-α, and **(F)** mRNA expression of interleukin 17.

IL-10 was adopted as the outcome measure in four studies. The results of the overall analysis showed that BBR could increase IL-10 levels in UC models [*n* = 92, SMD = 3.62, 95%CI (1.03, 6.22), *p* < 0.05; Figure 7A]. The heterogeneity test indicated the presence of heterogeneity (I^2^ = 93.1%, *p* < 0.01).

##### 3.4.5.2 Interleukin-1β

IL-1β was adopted as the outcome measure in eight studies. The results of the overall analysis showed that the BBR could reduce IL-1β levels in UC models [*n* = 162, SMD = −1.78, 95%CI (−2.58, −0.98), *p* < 0.05; Figure 7B]. The heterogeneity test indicated the presence of heterogeneity (I^2^ = 76.0%, *p* < 0.01).

##### 3.4.5.3 Interleukin 6

According to the data from the nine studies, the results of the overall analysis showed that the BBR could reduce IL-6 levels in UC models [*n* = 178, SMD = −1.78, 95%CI (−3.72, −1.68), *p* < 0.05; Figure 7C]. The heterogeneity test indicated the presence of heterogeneity (I^2^ = 82.2%, *p* < 0.01).

##### 3.4.5.4 Interferon-γ

The pooled data from five studies suggested that the BBR could reduce IFN-γ levels in UC models [*n* = 86, SMD = −1.84, 95%CI (−3.08, −0.59), *p* < 0.05; Figure 7D]. The heterogeneity test indicated the presence of heterogeneity (I^2^ = 79.3%, *p* < 0.01).

##### 3.4.5.5 Tumor necrosis factor α

The pooled data from ten studies suggested that the BBR could reduce TNF-α levels in UC models [*n* = 184, SMD = −3.78, 95%CI (−5.19, −2.37), *p* < 0.05; Figure 7E]. The heterogeneity test indicated the presence of heterogeneity (I^2^ = 87.8%, *p* < 0.01).

##### 3.4.5.6 mRNA level of interleukin 17

Only three studies adopted the mRNA level of interleukin 17 as an outcome measure. The results of the overall analysis showed that the BBR could reduce the mRNA level of interleukin 17 in UC models [*n* = 40, SMD = −5.08, 95%CI (−6.47, -3.69), *p* < 0.05; Figure 7F]. The heterogeneity test showed that there was no significant difference in heterogeneity among the studies (I^2^ = 31.2%, *p* = 0.234).

##### 3.4.5.7 Myeloperoxidase

**FIGURE 8:**
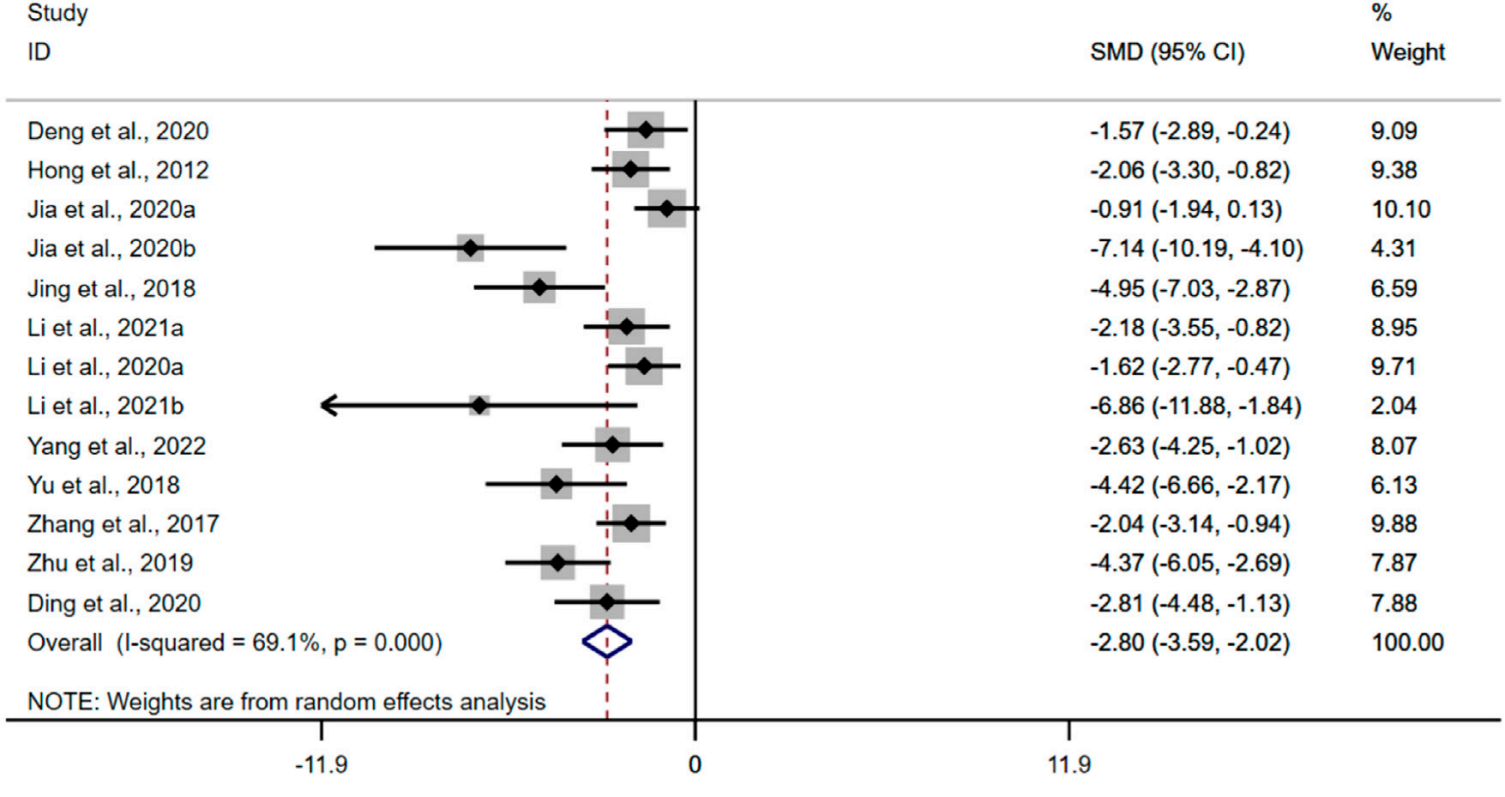
Forest plot: effect of berberine on MPO.

The pooled data from thirteen studies suggested that the BBR could reduce the activity of MPO in UC models [*n* = 186, SMD = −2.80, 95%CI (−3.59, −2.02), *p* < 0.05; Figure 8]. The heterogeneity test indicated the presence of heterogeneity (I^2^ = 69.1%, *p* < 0.01).

#### 3.4.6 Oxidative stress index changes

**FIGURE 9:**
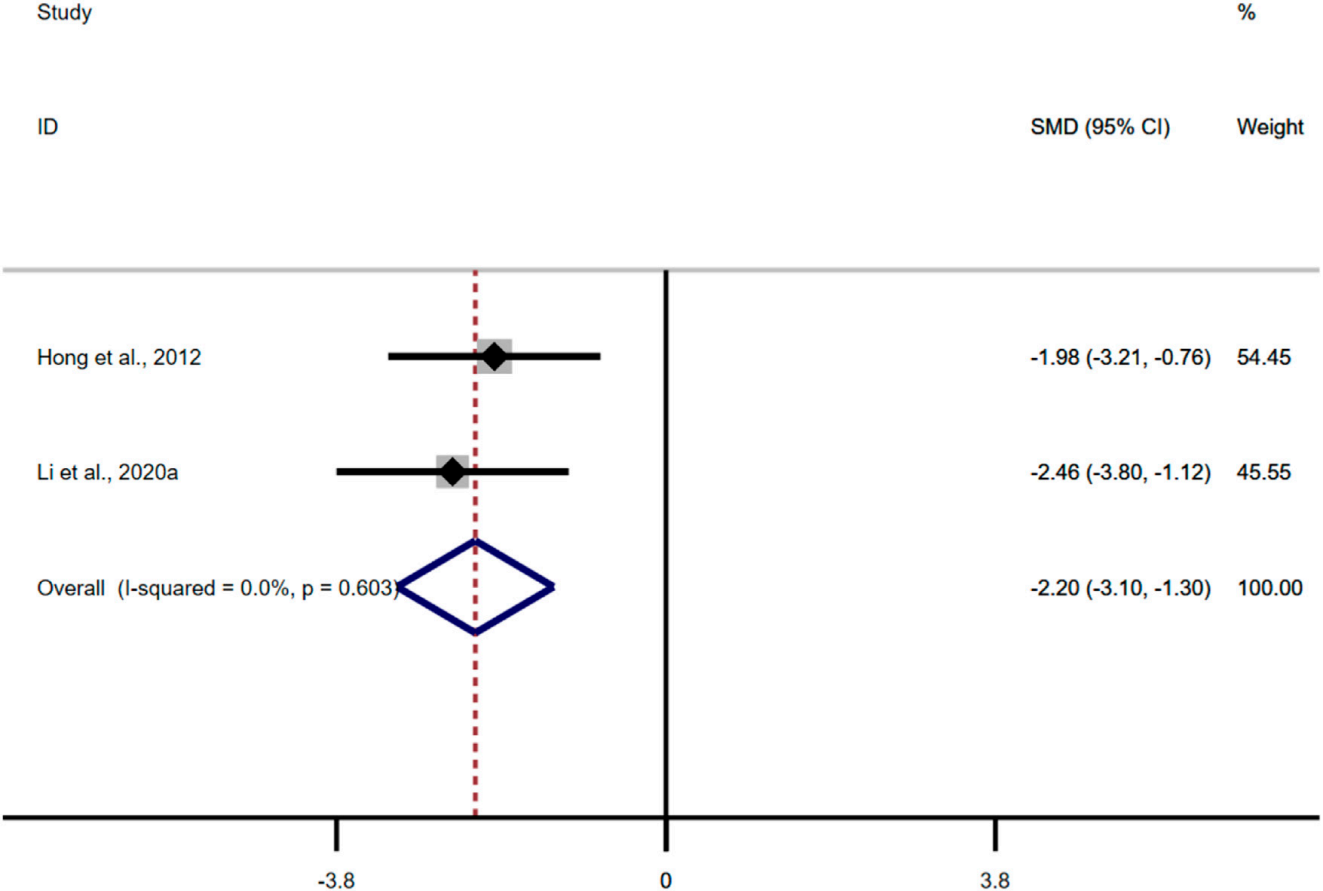
Forest plot: effect of berberine on MDA.

Only two studies adopted MDA as an outcome measure, and the results of the overall analysis showed that the BBR could reduce MDA levels in UC models [*n* = 86, SMD = −1.84, 95%CI (−3.08, −0.59), *p* < 0.05; Figure 9]. The heterogeneity test showed that there was no significant difference in heterogeneity among the studies (I^2^ = 0.0%, *p* = 0.603).

#### 3.4.7 Tight junction protein

**FIGURE 10:**
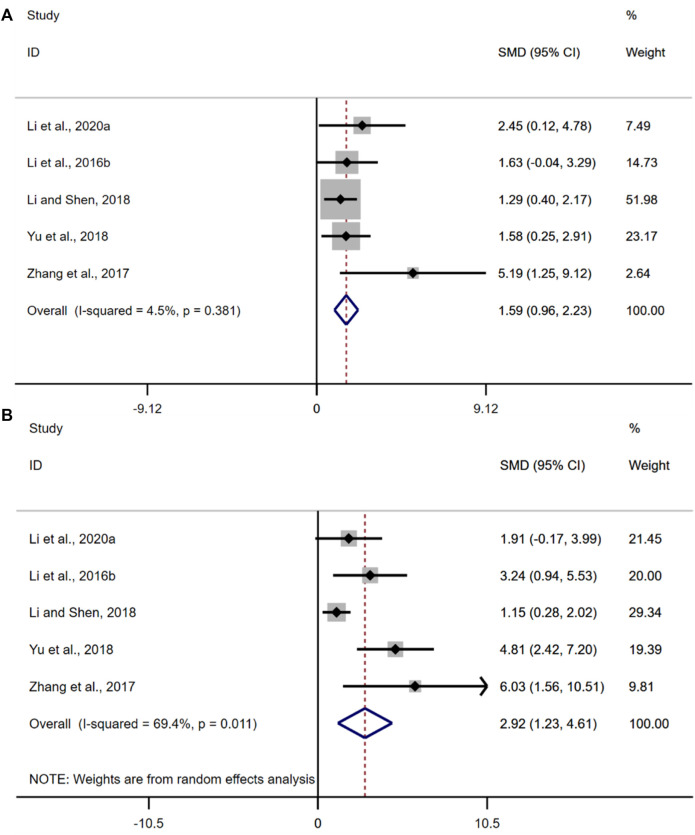
Forest plot: effect of berberine on **(A)** ZO-1, and **(B)** occludin.

A total of five studies were included in this analysis. The results of the overall analysis showed that the BBR could increase ZO-1 levels in UC models [*n* = 56, SMD = 1.59, 95%CI (0.96, 2.23), *p* < 0.05; Figure 10A]. The heterogeneity test showed that there was no significant difference in heterogeneity among the studies (I^2^ = 4.5%, *p* = 0.381). The results of the overall analysis showed that the BBR could increase occludin levels in UC models [*n* = 56, SMD = 2.92, 95%CI (1.23, 4.61), *p* < 0.05; Figure 10B]. The heterogeneity test indicated the presence of heterogeneity (I^2^ = 69.4%, *p* = 0.011).

### 3.5 Subgroup analysis

Because of the high heterogeneity among studies, we evaluated four subgroups of HCS, DAI, CL, and MPO in terms of animal species, animal sex, treatment cycles, and treatment dose. The results showed that treatment cycles may be the source of heterogeneity for HCS, treatment dose may be the source of heterogeneity for DAI, animal sex may be the source of heterogeneity for CL, and animal species, animal sex as well as treatment dose may be the source of heterogeneity for MPO. There was still significant heterogeneity among the studies. The results are presented in Supplementary Tables S2–S5.

### 3.6 Publication bias

**FIGURE 11:**
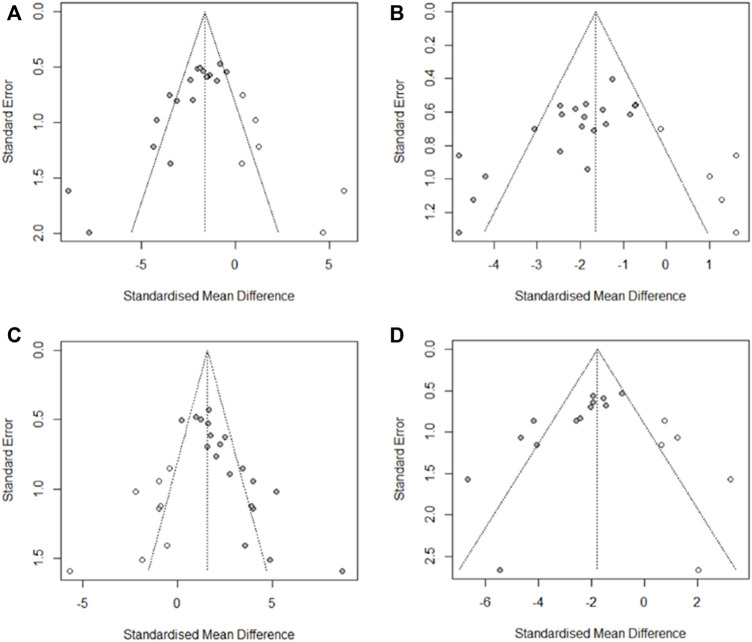
Results of trim and fill method. **(A)** DAI, **(B)** HCS, **(C)** CL, and **(D)** MPO.

We used Egger’s test to evaluate the publication bias of the four outcome indicators of DAI, CL, HCS, and MPO. The results showed publication bias for all four indicators. Asymmetries were then corrected for using the trim and fill method, respectively imputing six studies that may have been missed for DAI, respectively imputing six studies that may have been missed for HCS, respectively imputing eight studies that may have been missed for CL, and with respectively imputing five studies that may have been missed for MPO (Figure 11). The trim and fill analysis indicated that these missed studies didn’t change the magnitude of the overall pooled effect size (Supplemental Table S6).

### 3.7 Time-dose interval analysis

**FIGURE 12:**
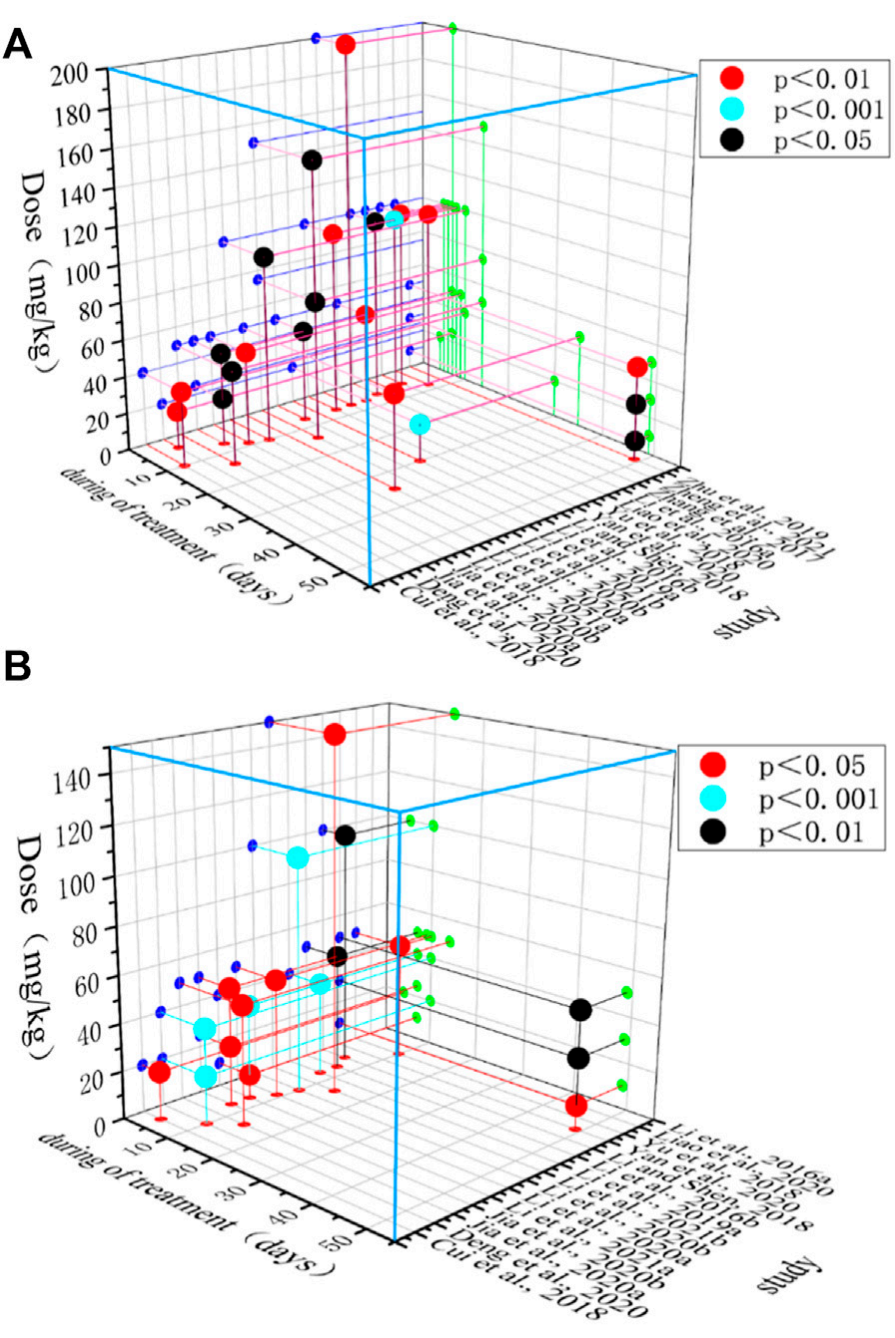
Time-dose interval analysis scatter plot. **(A)** HCS, and **(B)** MPO.

Endoscopic pathological findings can be used as an indication of eligibility for the treatment of UC (Mosli et al., 2017). MPO activity is proportional to colonic mucosal neutrophil infiltration and can be used as an indicator to evaluate the severity of UC (El Kebir et al., 2008). In this study, the time-dose effect relationship plots show that the minimum dose of BBR that improved HCS (*p* < 0.05) was 10 mg/kg and the maximum dose was 200 mg/kg. The minimum dose of BBR that improved MPO (*p* < 0.05) was 10 mg/kg and the maximum dose was 150 mg/kg. The minimum intervention period of BBR administration that improved HCS (*p* < 0.05) was 5 days and the maximum period was 49 days. The minimum intervention period of BBR administration that improved MPO (*p* < 0.05) was 4 days and the maximum period was 49 days. Overall results show that BBR at a dose of 10–150 mg/kg and with an intervention period of 5–49 days demonstrated relatively superior effectiveness. (Figure 12).

## 4 Discussion

### 4.1 Summary of evidence

To our knowledge, this is the first preclinical systematic review and meta-analysis to assess the pharmacological effects and potential mechanisms of BBR for the treatment of UC. We included 29 studies (508 experimental animals in total). We performed subgroup analyses for metrics with several included studies greater than ten. The results showed that treatment cycles could be the source of heterogeneity for HCS; treatment dose could be the source of heterogeneity for DAI; animal sex could be the source of heterogeneity for CL; animal species, animal sex, and treatment dose could be the source of heterogeneity for MPO. We performed a test for publication bias for four indicators with several studies greater than 10, and the results showed the presence of publication bias, but after the trim and fill method, there was no qualitative reversal in the results of the reanalysis, reflecting the stability of the results. Time-dose interval analysis showed that BBR at a dose of 10–150 mg/kg and with an intervention period of 5–49 days demonstrated relatively superior effectiveness. The results showed that in animal models of UC, BBR can protect the function of the colon and attenuate the pathological changes in the colonic tissue.

### 4.2 Possible protective mechanisms of BBR

The results of included studies suggest that the protective mechanisms of BBR in UC may include the following aspects.

**FIGURE 13:**
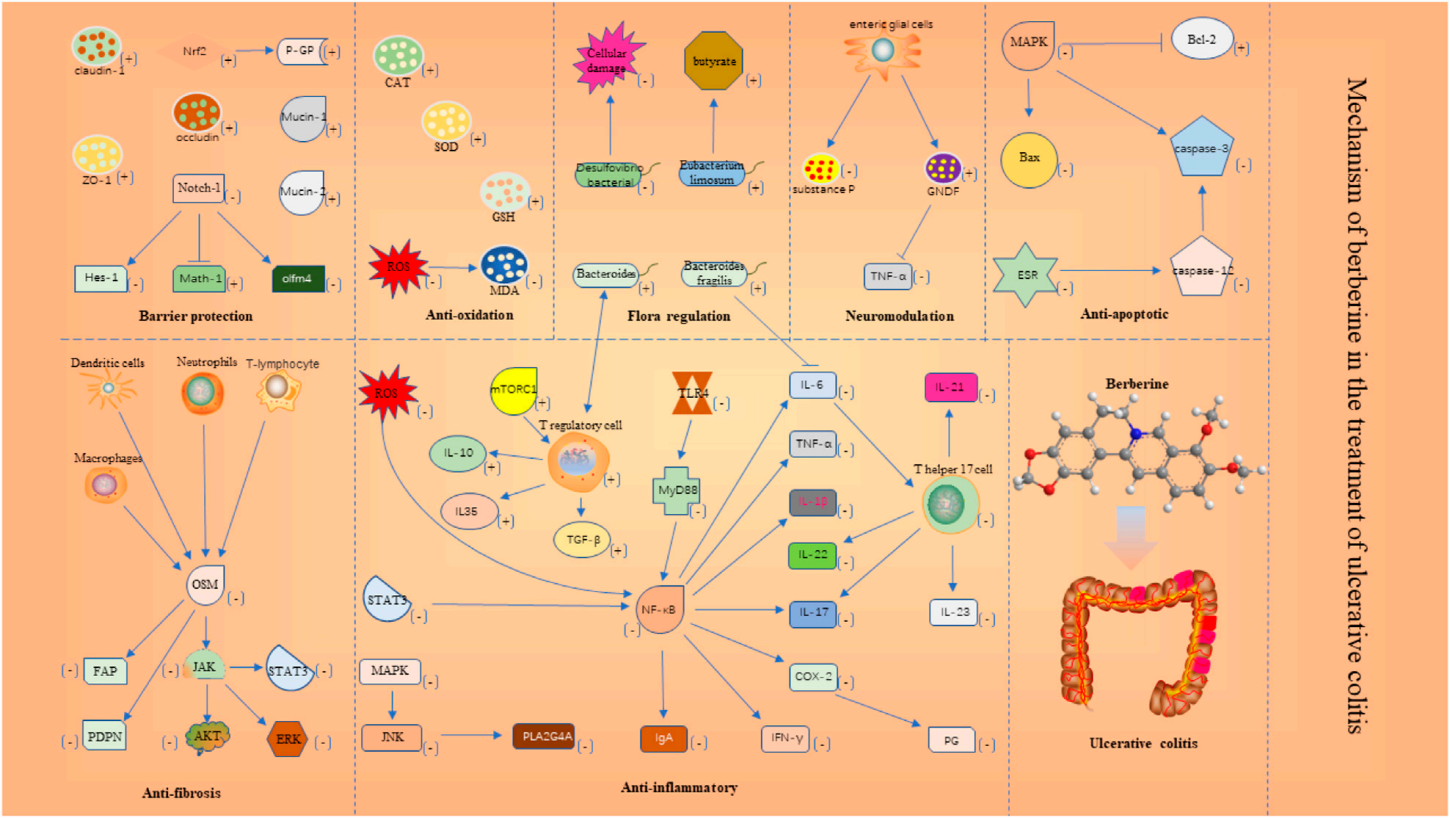
Mechanism of berberine in the treatment of ulcerative colitis.

(1) Antioxidant. Oxidative stress is closely linked to the pathogenesis of UC. Superoxide dismutase (SOD) and catalase (CAT) and other enzymes that build cellular antioxidant defense systems can protect cells (Akateh et al., 2019). Reactive oxygen species (ROS)-mediated protein modifications and MDA production from lipid peroxidation cause self-tolerance disruption (Frostegard et al., 2005; Wang et al., 2008; Wang et al., 2012). Experimental data suggested that the BBR can increase the expression levels of SOD and CAT antioxidant factors, and reduce the MDA and ROS.
(2) Anti-apoptotic. Inhibition of apoptosis plays an important role in UC. Caspase-3 degrades proteins and plays a major role in cell death (Kaur et al., 2019). The B-cell lymphoma/leukemia-2 gene (Bcl-2) regulates calcium ions to prevent cell death (Distelhorst, 2018). The release of BCL2-Associated X (BAX) can induce cell death (Lin et al., 2019). Our findings showed that the BBR can reduce Caspase-3 and BAX production and elevate Bcl-2 levels in the colonic mucosa by inhibiting MAPK activation and inhibiting the endoplasmic reticulum stress (ERS)-induced Caspase-12/Caspase-3 process.
(3) Neuromodulation. Neuromodulation is also an important part of UC recovery. Substance P has pro-inflammatory effects on immune and epithelial cells and is involved in inflammation of the gastrointestinal tract (O'Connor et al., 2004). Glial cell line-derived Neurotrophic Factor (GNDF) regulates the development of the peripheral nervous system (Malin and Davis, 2008). Our findings showed that the BBR can regulate the secretion of neuropeptides by mucosal intestinal glial cells, such as inhibiting the secretion of P substances, promoting the secretion of GNDF to regulate the relationship between enteric glial cells and immune cells and epithelial tight junctions.
(4) Anti-fibrotic. Patients with UC are often associated with submucosal fibrosis. Oncostatin M (OSM), synthesized mainly by activated macrophages, neutrophils, dendritic cells, and T cells, is a member of the IL-6 cytokine family, and excess OSM further leads to massive inflammatory cell infiltration into the mucosa, pathologically leading to intestinal fibrosis (Richards, 2013; Hermanns, 2015; West et al., 2017). Increased OSM leads to fibroblast activation protein (FAP) and podoplanin (PDPN) overexpression in OSMR + mesenchymal cells and promotes intestinal fibrosis in patients (West et al., 2017). Our findings showed that the BBR can inhibit the expression of FAP and PDPN by suppressing the production of OSM, which in turn inhibits the activation of OSMR + stromal cells, or by directly inhibiting the response of OSM to OSMR + stromal cells. In addition, the BBR can simultaneously interfere with JAK-STAT, ERK, and AKT signaling pathways in OSMR + stromal cells.
(5) Anti-inflammatory. Intestinal inflammation is closely linked to the pathogenesis of UC. Nuclear factor kappa-B (NF-κB) translocates from the cytoplasm to the nucleus to bind DNA and promote the production of pro-inflammatory mediators and chemokines (Wang et al., 2020), such as TNF-α, IL-1β, and IL-6, IL-17, IFN-γ, IgA, and cyclooxygenase-2 (COX-2). COX is a key rate-limiting enzyme for the synthesis of prostaglandins (PG) (Steiner et al., 2006). PG is the mediator of the inflammatory response (Post et al., 1990). T helper 17 cells (Th17) produce IL-17, IL-21, IL-22, IL-23, and IL-25, which are pro-inflammatory cytokines (Korn et al., 2009; Weaver et al., 2013). T regulatory cells (Treg) secret the inflammatory suppressive cytokines IL-10, IL35, and TGF-β (Jonuleit and Schmitt, 2003). Our findings showed that the BBR can inhibit TLR4/MyD88/NF-κB signaling pathway; inhibit NF-κB translocation from cytoplasm to nucleus; inhibit NF-κB activation by ROS; block STAT3/NF-κB signaling pathway; block NF-κB/COX-2 signaling pathway to reduce PG. In addition, the BBR can inhibit PLA2G4A to suppress the expression of pro-inflammatory genes, and the BBR can decrease the number of Th17 and activate the mTORC1 pathway to increase the number of Treg.
(6) Barrier protection. The integrity of the intestinal mucosa is important for the healing of UC. Notch-1 is mainly distributed in intestinal epithelial cells (Siebel and Lendahl, 2017). Notch-1 regulates the proliferation and differentiation of colonic epithelial cells through its downstream Hes-1, Math-1, and olfm4 (Zheng et al., 2011). Mucin 2, expressed primarily in cup cells, provides broad protection and maintains intestinal integrity (Gundamaraju and Chong, 2021). Mucin-1 is a cell surface mucin that is an important barrier in the gastrointestinal tract (Ham and Kaunitz, 2008). P-glycoprotein (P-GP) has a role in maintaining the barrier function of intestinal mucosal epithelial cells (Kodan et al., 2021). Occludin, claudin-1, and ZO-1 play a key role in regulating the selectivity and permeability of the intestinal wall. Our findings showed that the BBR can inhibit the expression of Notch-1 receptor and reduce the expression of Hes-1 protein, reduces olfm4 target protein, and promotes the expression of Math-1 while increasing the expression of ZO-1, occludin, claudin-1, and mucin-1 and mucin-2. In addition, the BBR can activate nuclear factor erythroid-2 related factor 2 (Nrf2) translocation into the nucleus to promote the expression of P-GP.
(7) Flora regulation. The stability of the intestinal flora is also important for the recovery of UC. *Mycobacterium avium* can interact with Treg and promote IL-10 production by producing metabolites (Smith et al., 2013). Eubacterium limosum can improve mucosal integrity by producing butyrate (Kanauchi et al., 2006). In UC, the increase of *Vibrio* desulfuricans species is harmful to colonic epithelial cells (Rowan et al., 2010). Our findings showed that the BBR reduced the abundance of Desulfovibrio and increased the abundance of Eubacterium strains and *Bacteroides*. In addition, the BBR can promote the growth of *Bacteroides fragilis* and inhibit IL-6 secretion, which in turn inhibits the differentiation of Th17 cells. Details are shown in Figure 13.

### 4.3 Implications

UC is an idiopathic inflammatory disease affecting the large intestine, the exact etiology, and pathogenesis of which are still unknown. Currently, there are no established and effective treatments for UC. To date, systematic evaluation of UC is limited, and there is a lack of evidence of effective drugs in preclinical animal studies. The main reason for this phenomenon is the differences in the index systems of these studies. In this paper, we synthesize the therapeutic studies of BBR in UC to provide a reference for further clinical studies. The results showed that the BBR can reduce DAI scores, alleviate UC-induced CL loss, prevent weight loss, and reduce HCS. Mechanistically, BBR can reduce MPO activity and MDA levels, reduce levels of pro-inflammatory factors IL-1β, IL-6, TNF-α, IFN-γ, and mRNA expression of IL-17, increase levels of anti-inflammatory factor IL-10, and increase levels of ZO-1 and occludin.

### 4.4 Methodological deficiencies and suggestions

To date, preclinical studies using animal studies to verify the therapeutic effects of drugs remain an important and indispensable part of drug development. Preclinical animal studies suffer from methodological problems such as a high risk of bias and rigorous rationalization of experimental design, accompanied by low reproducibility of experimental results. And many drugs have good pharmacological effects in animal models, but clinical conversion rates are generally low (van der Worp and Sandercock, 2012). This affects the motivation of scientific research to some extent. The systematic evaluation in this paper has the following limitations: 1) Our current search included research literature in English, which leads to a certain degree of language bias. Researchers around the world have been researching natural products. Traditional Chinese medicine has a long history of existence and efficacy. However, research in Chinese medicine is currently limited to Asian countries such as China, Japan, India, and Korea. 2) There is a publication bias in the literature included in this systematic evaluation, which may be related to some extent to the quality of the included studies. No studies reflected specific randomization methods, although the use of randomization was partially described. No studies mentioned allocation concealment and blinding of experimenters. Fewer studies communicated the use of blinding for outcome analysts. 3) There was heterogeneity in most of the indicators evaluated in this system. Heterogeneity was reduced in some studies under subgroup analyses that we set up in advance, however, most studies were not analyzed for sources of heterogeneity. It is speculated that the heterogeneity may originate from the differential methodological design, such as the differences in feeding environment, and criteria of the modeling approach. However, since, the data for this evaluation were not raw data and were derived from electronic data calipers, this may have contributed to some extent to the error in the results. 4) The results of the systematic review in this paper lacked toxicological information. None of the included studies had rigorous toxicology reports. Only a very few studies reported aspartate aminotransferase and alanine aminotransferase, but this is still not a true toxicology report. Recommendation: A uniform and complete experimental protocol should be established. Negative results were not demonstrated in this included study, and it is hoped that researchers will report negative results truthfully and that negative results will be more widely accepted. The reasonable uniformity of the ending index detection and calculation methods, as well as the presentation of the original data, can also reduce to a certain extent the accuracy of the later evaluation by other researchers and reduce the bias of the results.

## 5 Conclusion

The current meta-analysis showed that BBR can reduce DAI scores, HCS, and levels of pro-inflammatory factors while increasing the levels of tight junction proteins. The underlying mechanisms of these protective effects involve antioxidant, anti-apoptotic, neuromodulation, anti-fibrotic, anti-inflammatory, barrier protection, and flora regulation aspects. The BBR at a dose of 10–150 mg/kg with an intervention period of 5–49 days demonstrated relatively superior effectiveness. However, additional attention should be paid to these outcomes due to the heterogeneity and methodological quality of the studies. Future more rigorous experimental designs and more comprehensive studies are needed to test the protective effects of BBR on UC.

## Data Availability

The original contributions presented in the study are included in the article/Supplementary Material, further inquiries can be directed to the corresponding authors.
