# Supplementary material for: Pharmacological effects of berberine on models of ulcerative colitis: A meta-analysis and systematic review of animal studies
Source: Front Pharmacol. 2022 Sep 6;13:937029. doi: 10.3389/fphar.2022.937029 (PMC9486070; doi:10.3389/fphar.2022.937029)
Supplement: Supplementary file 1 [file DataSheet1.docx]

**Supplementary Table 1**

Literature search strategy for berberine in the treatment of ulcerative colitis

| Search Strategy (PubMed) | |
| --- | --- |
| #1 | Idiopathic Proctocolitis [Mesh] |
| #2 | Ulcerative Colitis [Title/Abstract] |
| #3 | Colitis Gravis [Title/Abstract] |
| #4 | Inflammatory Bowel Disease, Ulcerative Colitis Type [Title/Abstract] |
| #5 | Colitis, Ulcerative [Title/Abstract] |
| #6 | #1 or #2 or #3 or #4 or #5 |
| #7 | Berberine [Mesh] |
| #8 | Umbellatine [Title/Abstract] |
| #9 | Berbines [Title/Abstract] |
| #10 | Dioxolanes [Title/Abstract] |
| #11 | Dioxoles [Title/Abstract] |
| #12 | #7 or #8 or #9 or #10 or #11 |
| #13 | #6 and #12 |

**Supplementary Table 2** The subgroup analyses of HCS

| **Parameter** | **Subgroup** |  | **No. of studies** | **SMD [95% CI]** | **I^2^ (%)** | **P for heterogeneity** |
| --- | --- | --- | --- | --- | --- | --- |
| HCS | species | mice | 16 | -2.15[-2.65, -1.65] | 58.3 | P< 0.01 |
|  |  | RAT | 3 | -3.56[-5.57, -1.56] | 76.5 | P< 0.05 |
|  | sex | Female | 3 | -1.79[-3.18, -0.39] | 73.2 | P< 0.05 |
|  |  | No mention | 2 | -1.71[-3.41, 0.00] | 65.1 | P> 0.05 |
|  |  | male | 14 | -2.55[-3.13, -1.97] | 61.8 | P< 0.01 |
|  | treatment cycles（days） | ≤10 | 14 | -2.63[-3.30, -1.96] | 68.3 | P< 0.01 |
|  |  | >10 | 5 | -1.61[-2.11, -1.11] | 0 | P> 0.05 |
|  | treatment dose(mg/kg) | ≤50 | 11 | -2.16[-2.80, -1.52] | 59.6 | P< 0.01 |
|  |  | >50 | 8 | -2.55[-3.35, -1.75] | 66 | P< 0.01 |

**Supplementary Table 3** The subgroup analyses of DAI

| **Parameter** | **Subgroup** |  | **No. of studies** | **SMD [95% CI]** | **I^2^ (%)** | **P for heterogeneity** |
| --- | --- | --- | --- | --- | --- | --- |
| DAI | species | mice | 15 | -2.03[-2.60, -1.47] | 65.3 | P< 0.01 |
|  |  | RAT | 3 | -5.34[-8.22, -2.47] | 82.1 | P< 0.01 |
|  | sex | Female | 3 | -1.75[-3.15, -0.34] | 73.3 | P< 0.05 |
|  |  | No mention | 1 | -4.72[-7.08, -2.36] | - | - |
|  |  | male | 14 | -2.59[-3.36, -1.83] | 77.4 | P< 0.01 |
|  | treatment cycles（days） | ≤10 | 12 | -2.25[-2.89, -1.60] | 68 | P< 0.01 |
|  |  | >10 | 6 | -3.64[-5.55, -1.73] | 86.4 | P< 0.01 |
|  | treatment dose(mg/kg) | ≤50 | 12 | -2.98[-4.01, -1.95] | 82.3 | P< 0.01 |
|  |  | >50 | 6 | -1.95[-2.59, -1.31] | 44.5 | P> 0.05 |

**Supplementary Table 4** The subgroup analyses of CL

| **Parameter** | **Subgroup** |  | **No. of studies** | **SMD [95% CI]** | **I^2^ (%)** | **P for heterogeneity** |
| --- | --- | --- | --- | --- | --- | --- |
| CL | species | mice | 17 | 2.53[1.88, 3.18] | 72.7 | P< 0.01 |
|  |  | RAT | 2 | 5.88[-0.98, 12.15] | 93.5 | P< 0.01 |
|  | sex | Female | 4 | 2.46[1.72, 3.20] | 8.5 | P> 0.05 |
|  |  | No mention | 2 | 3.47[2.12, 4.82] | 0 | P> 0.05 |
|  |  | male | 13 | 2.81[1.90, 3.73] | 82.5 | P< 0.01 |
|  | treatment cycles（days） | ≤10 | 12 | 2.56[1.87, 3.25] | 67.1 | P< 0.01 |
|  |  | >10 | 7 | 3.36[1.75, 4.98] | 86.5 | P< 0.01 |
|  | treatment dose(mg/kg) | ≤50 | 12 | 2.88[2.00, 3.77] | 74.8 | P< 0.01 |
|  |  | >50 | 7 | 2.62[1.44, 3.80] | 82.7 | P< 0.01 |

**Supplementary Table 5** The subgroup analyses of MPO

| **Parameter** | **Subgroup** |  | **No. of studies** | **SMD [95% CI]** | **I^2^ (%)** | **P for heterogeneity** |
| --- | --- | --- | --- | --- | --- | --- |
| MPO | species | mice | 10 | -2.09[-2.67, -1.51] | 38.3 | P> 0.05 |
|  |  | RAT | 3 | -5.07[-6.43, -3.71] | 18.4 | P> 0.05 |
|  | sex | Female | 3 | -2.41[-3.26, -1.56] | 0 | P> 0.05 |
|  |  | No mention | 1 | 3.47[2.12, 4.82] | - | - |
|  |  | male | 9 | -3.27[-4.44, -2.09] | 78.3 | P< 0.01 |
|  | treatment cycles（days） | ≤10 | 10 | -2.72[-3.49, -1.95] | 60.2 | P< 0.01 |
|  |  | >10 | 3 | -3.47[-6.64, -0.29] | 87.2 | P< 0.01 |
|  | treatment dose(mg/kg) | ≤50 | 10 | -2.99[-3.96, -2.02] | 73.3 | P< 0.01 |
|  |  | >50 | 3 | -2.15[-3.46, -0.84] | 5.02 | P> 0.05 |

**Supplementary Table 6** Results from Egger's test and trim and fill analysis

| **Outcomes** | **Egger's test P value** | **Before trim and fill** | | | **after trim and fill** | | |
| --- | --- | --- | --- | --- | --- | --- | --- |
|  |  | P value | SMD (random) | Number  of studies | P value | SMD (random) | Number of studies |
| DAI | P< 0.0001 | 0.000 | -2.513 | 18 | 0.0027 | -1.6009 | 24 |
| HCS | P= 0.0006 | 0.000 | -2.830 | 19 | 0.000 | -1.6427 | 25 |
| CL | P< 0.0001 | 0.000 | 2.763 | 19 | 0.0011 | 1.5803 | 27 |
| MPO | P= 0.0003 | 0.000 | -2.80 | 13 | 0.0006 | -1.7779 | 18 |
